# The use of innominate artery cannulation for antegrade cerebral perfusion in aortic dissection

**DOI:** 10.1186/s13019-020-01249-1

**Published:** 2020-07-31

**Authors:** Eden C. Payabyab, Jonathan M. Hemli, Allan Mattia, Alex Kremers, Sohrab K. Vatsia, S. Jacob Scheinerman, Efstathia A. Mihelis, Alan R. Hartman, Derek R. Brinster

**Affiliations:** 1 Division of Cardiac Surgery, Virginia Commonwealth University Health Systems, Richmond, VA UK; 2 Department of Cardiothoracic Surgery, New York Presbyterian-Weill Cornell Medicine, New York, NY USA; 3 Department of Cardiovascular and Thoracic Surgery, Lenox Hill Hospital / Northwell Health, New York, NY USA; 4 Department of Cardiovascular and Thoracic Surgery, North Shore University Hospital / Northwell Health, Manhasset, NY USA; 5 Rush University, Chicago, IL USA

**Keywords:** Aortic dissection, Aortic arch, Direct innominate cannulation, Cerebral perfusion, Outcomes

## Abstract

**Background:**

Direct cannulation of the innominate artery for selective antegrade cerebral perfusion has been shown to be safe in elective proximal aortic reconstructions. We sought to evaluate the safety of this technique in acute aortic dissection.

**Methods:**

A multi-institutional retrospective review was undertaken of patients who underwent proximal aortic reconstruction for Stanford type A dissection between 2006 and 2016. Those patients who had direct innominate artery cannulation for selective antegrade cerebral perfusion were selected for analysis.

**Results:**

Seventy-five patients underwent innominate artery cannulation for ACP for Stanford Type A Dissections. Isolated replacement of the ascending aorta was performed in 36 patients (48.0%), concomitant aortic root replacement was required in 35 patients (46.7%), of whom 7 had a valve-sparing aortic root replacement, ascending aorta and arch replacement was required in 4 patients (5%). Other procedures included frozen elephant trunk (*n* = 11 (14.7%)), coronary artery bypass grafting (*n* = 20 (26.7%)), and peripheral arterial bypass (*n* = 4 (5.3%)). Mean hypothermic circulatory arrest time was 19 ± 13 min. Thirty-day mortality was 14.7% (*n* = 11). Perioperative stroke occurred in 7 patients (9.3%).

**Conclusions:**

This study is the first comprehensive review of direct innominate artery cannulation through median sternotomy for selective antegrade cerebral perfusion in aortic dissection. Our experience suggests that this strategy is a safe and effective technique compared to other reported methods of cannulation and cerebral protection for delivering selective antegrade cerebral perfusion in these cases.

## Background

Stanford type A dissections carry a high mortality with reports ranging from 17 to 26% [1–4]. Timely operative intervention improves outcomes with delays increasing mortality 1–2% every hour in the first 48 h. Repair of the aortic dissection requires complex circulatory management and cerebral protection during circulatory arrest. Strategies to improve outcomes include hypothermia alone or in conjunction with antegrade cerebral perfusion (ACP) or retrograde cerebral perfusion (RCP). Moderate hypothermia with ACP has been shown to be a safe and effective strategy from neuroprotection in aortic arch reconstruction including operative interventions for aortic dissections [5–8].

Several techniques for administering selective ACP (SACP) have been described including right axillary artery cannulation with concomitant occlusion of the base of the innominate artery [9], direct placement of balloon-tipped catheters into the ostia of the arch vessels [10], and cannulation of the innominate artery via a side-graft [11, 12]. Neurologic events with these techniques range from 3.4% in elective aortic arch operations to 12% in acute Stanford type A dissections. An alternative technique for SACP, utilizing direct innominate artery cannulation, has been shown to be safe in elective arch reconstruction with reported stroke and mortality rates of 1% [13, 14].

The outcomes of direct innominate artery cannulation for SACP in acute aortic dissection have yet to be reported. We sought to evaluate the safety and efficacy of this technique in acute Stanford type A dissections.

## Methods

### Patients

We performed a multi-institutional comprehensive review of all patients who underwent repair of Stanford type A dissection between 2006 and 2016. Seventy-five patients had direct cannulation of the innominate artery for SACP during their dissection repair. The study protocol was approved by the institutional board reviews of the Northwell Health System and Virginia Commonwealth University Health System.

### Surgical technique

**Fig. 1:**
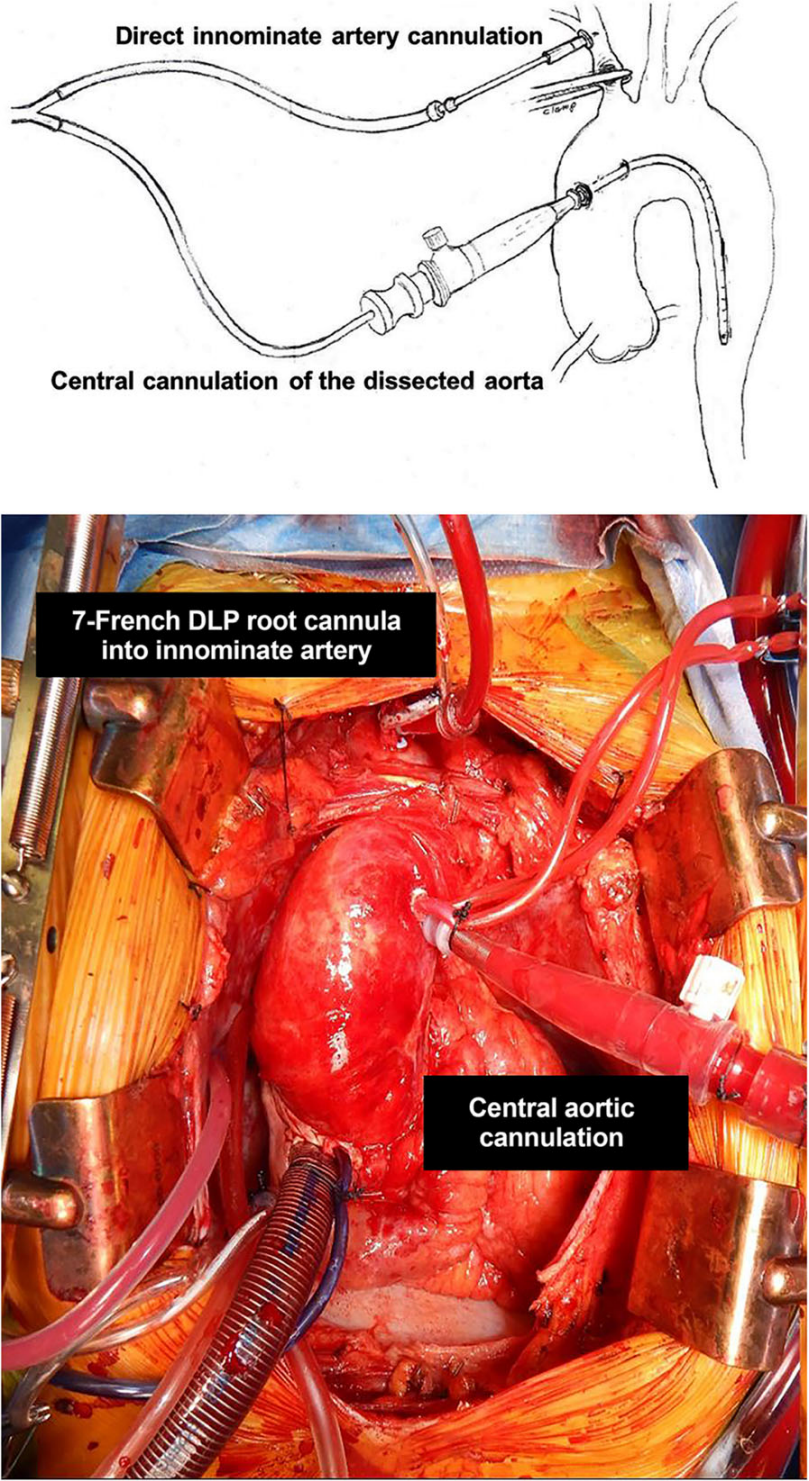
Direct cannulation of innominate artery with 7-French standard-tip DLP aortic root cannula

**Fig. 2:**
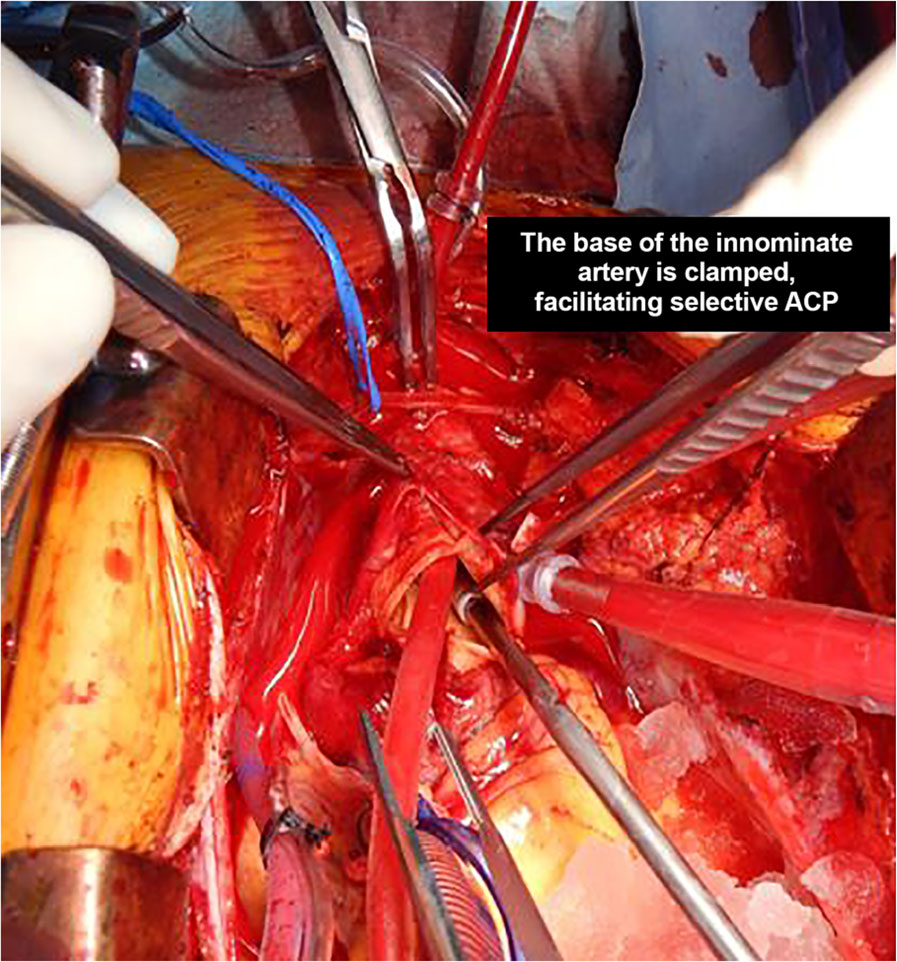
The origin of the left common carotid artery is also isolated and clamped, keeping the Circle of Willis pressurized and thereby preventing a ‘steal’ phenomenon

The dissected ascending aorta is cannulated directly to initiate cardiopulmonary bypass utilizing transesophageal echo guidance to place a long percutaneous arterial cannula placed with Seldinger technique as previously described [15]. The innominate artery is then cannulated directly with a 7-French standard-tip DLP aortic root cannula and connected to the arterial limb of the cardiopulmonary bypass circuit utilizing standard 3/8″ tubing, with a customized 1/4″ branched limb that has a perfusion adapter to attach to the innominate artery cannula. Figure 1*.* Once the patient’s core temperature reaches the desired target (typically moderate hypothermia at 28 °C), the base of the innominate artery is clamped proximal to the innominate artery cannulation site. The origin of the left common carotid artery is also isolated and clamped, keeping the Circle of Willis pressurized and thereby preventing a ‘steal’ phenomenon. Figure 2*.* Cerebral perfusion is measured by an invasive arterial line situated in the right radial artery, and adjusted according to arterial pressure and continuous non-invasive monitoring of cerebral oxygen saturations using near-infrared spectroscopy.

## Results

Preoperative patient demographics are presented in Table 1. Operative data including details of proximal aortic reconstruction, concomitant procedures and intraoperative times are described in Table 2. Perioperative outcomes are listed in Table 3.

**Table 1:** Patient Demographics

Patient Demographics	
*Variable*	*n (%)*
Female gender	28 (37.3)
Age, years (mean ± SD)	58.9 ± 15.8
BMI, kg/m^2^ (mean ± SD)	29.9 ± 9.6
Hypertension	37 (49.3)
History of smoking	35 (46.7)
Dyslipidemia	25 (33.3)
Heart failure	10 (13.3)
Cerebrovascular disease	10 (13.3)
Peripheral vascular disease	8 (10.7)
History of prior MI	8 (10.7)
Chronic lung disease	8 (10.7)
Marfan syndrome	1 (1.3)

*SD* Standard deviation, *MI* Myocardial infarction

**Table 2:** Operative Data

Operative Data	
*Variable*	*n (%)*
Proximal aortic reconstruction	
Isolated replacement of ascending aorta	36 (48.0)
Ascending Aorta and arch replacement	4 (5)
Aortic root replacement	35 (46.7)
Composite valve-graft conduit	32 (42.7)
Valve-sparing	3 (4.0)
Total arch replacement	7 (9.3)
Concomitant procedures	
Frozen elephant trunk	11 (14.7)
CABG	20 (26.7)
Peripheral arterial bypass	4 (5.3)
Cardiopulmonary bypass time, minutes (mean ± SD)	166.1 ± 71.9
Aortic cross-clamp time, minutes (mean ± SD)	108.1 ± 47.7
Circulatory arrest time, minutes (mean ± SD)*	19.2 ± 13.0
Lowest intraoperative temperature, °C (mean ± SD)	24.8 ± 11.1

*CABG* Coronary artery bypass grafting, *SD* Standard deviation

**Table 3:** Perioperative Outcomes

Perioperative Outcomes	
*Variable*	*n (%)*
Stroke	7 (9.3)
Re-operation for bleeding	6 (8.0)
Perioperative MI	1 (1.3)
Deep sternal wound infection	2 (2.7)
New renal failure requiring dialysis	11 (14.7)
Prolonged intubation	37 (49.3)
Tracheostomy	5 (6.7)
Multi-system organ failure	8 (10.7)
Limb ischemia	4 (5.3)
Postop length of stay, days (median ± SD)	10.0 ± 9.1
30-day mortality	11 (14.7)

*MI* Myocardial infarction, *SD* Standard deviation.

Perioperative stroke occurred in 7 patients (9.3%). Four patients experienced neurological deficits including dysphagia and motor dysfunction with complete resolution of symptoms within 30 days. The remaining three patients (4%), who presented in extremis, unable to asses neurologic exam and found to have hemopericardium, had no improvement in neurologic status resulting in poor prognosis and family withdrawal of care. In the patients presenting to the operating room with no neurologic deficits and clinically intact there was 5.3% postoperative neurological complication rate.

Perioperative mortality was 14.7% (11 patients) which included 3 patients who experienced a neurological complication. Of our perioperative mortalities, 5 patients presented in extremis. Amongst the 6 patients presenting hemodynamically stable the postoperative mortality was 8%. (Intraoperative death occurred in three patients, with one unable to wean from bypass and two experiencing uncontrollable hemorrhage. These patients were hypotensive on presentation, with two requiring CPR and all found to have hemopericardium. A 63-year-old male who underwent an ascending arch, aortic root replacement, hemiarch and CABG required ECMO for ventricular assistance secondary to ventricular fibrillation. He required a re-exploration on postoperative day one for bleeding, required a left ventricular repair, and interventions for ventricular fibrillation. Given the likely poor outcome the patient’s family withdrew care. A 51-year-old male that presented with malperfusion and Type I dissection underwent replacement of ascending aorta with resuspension of the aortic valve, hemi-arch replacement, placement of descending thoracic aortic stent graft and ascending aorta to left femoral artery bypass. Postoperatively the patient required continued administration of blood products and vasopressors due to coagulopathic bleeding and hypotension. The patient experienced multisystem organ failure leading to death. A 79-year-old female underwent a complex aortic root replacement, ascending aorta and proximal arch replacement and a 2 vessel CABG who experienced disseminated intravascular coagulopathy and postoperative liver failure leading to multiorgan system failure and eventual death.

## Discussion

Early mortality in patients undergoing surgical repair of type A aortic dissection is reported as high as 31%. (3) Developing an efficient and safe surgical technique to cannulate and provide cerebral perfusion is essential to successfully perform a repair of type A aortic dissection pathology. Several cannulation techniques have been described for the use of arterial inflow in the surgical repair of type A aortic dissections, all with potential benefits and drawbacks. Femoral artery cannulation, carries the potential complication of cerebral embolization and organ malperfusion. The use of the axillary artery for arterial inflow via a side-graft or direct cannulation has the disadvantage of needing a second incision and the additional time to cannulate the axillary artery [9, 16]. Direct cannulation of the innominate artery for full cardiopulmonary bypass is an alternative cannulation site described [11, 13, 17]. Preventza et al describe innominate artery cannulation with the use of a side graft as an alternate technique to peripheral cannulation for surgical repair, having a low stroke and mortality rate [12]. An advantage of these techniques in the use of these sites for SACP during circulatory arrest. The use of central cannulation has also been shown to be safe in the surgical repair of type A aortic dissections [15, 18, 19].

Evaluation of the different cannulation strategies by various groups show similar outcomes. Kamiya et al [20] reviewed 235 patients who underwent operative intervention for type A aortic dissection. They compared the patients who underwent cannulation of the ascending aorta and femoral artery and found no difference in long-term outcomes between the two groups. Stamou et al [21] compared early postoperative outcomes in 305 patients at multiple institutions who underwent axillary versus femoral cannulation over ten years. They found no difference in operative mortality.

The use of antegrade cerebral perfusion (ACP) has been shown to reduce neurologic morbidity after hypothermic circulatory arrest in proximal aortic reconstruction [22, 23]. Several techniques for administering selective ACP have been described including right axillary cannulation with concomitant occlusion of the base of the innominate artery, direct placement of balloon tip catheters into the ostia of the arch vessels under direct vision after circulatory arrest and cannulation of the innominate artery after circulatory arrest via a side-graft. Neurologic events with these techniques are reported to be up 12% in acute type A dissections [9, 10, 24]. Our use of direct innominate artery cannulation for SACP (9.3%) are similar. Of the 7 patients, 3 presented in extremis unable to be evaluated neurologically. In evaluating patients who presented neurologically intact, our neurologic events (4%) are decreased compared to other techniques.

An alternate technique for SACP, utilizing direct innominate artery cannulation has been described. Garg et al [25] describe a technique in which central aortic cannulation for elective aortic surgery. is performed followed by direct innominate artery cannulation with a 14F pediatric venous cannula for SACP after hypothermia. They reported outcomes of 50 patients who underwent replacement of the ascending aorta using an open distal anastomosis or hemiarch replacement. The operative mortality was 2% with a stroke rage of 2%. A similar technique for elective aortic surgery described by Jassar et al [13] utilizes direct cannulation of the innominate artery for SACP. Their technique includes arterial cannulation of the ascending aorta and use of a short tipped 9-Fr cardioplegia catheter for direct innominate cannulation following hypothermia and circulatory arrest. Our method is similar apart from their use of a larger cannula to directly cannulate the innominate artery. They evaluated 100 elective hemiarch reconstructions with results that showed a 30-day in-hospital mortality and stroke rate of 1%.

## Conclusions

The use of direct innominate artery cannulation with an accessory cannula for SACP in elective ascending aortic repairs is comparable to alternative methods. To our knowledge the use of this method in acute type A aortic dissection have yet to be reported. We performed a multi-institutional review of 75 patients over ten years. All these patients underwent direct cannulation of the innominate artery with a 7-French standard-tip DLP aortic root cannula. Our patient cohort included seven patients who presented with extension of the dissection into the innominate artery, which did not preclude the use of the technique. Our 30-day mortality was 14.7% and a perioperative stroke rate of 9.3%. These outcomes compare to those reported in other contemporary series of acute dissection repair, including IRAD data.

Our study has limitations of being a retrospective and non-comparative review. The experience is multi-intuitional but is limited to a single surgeon experience. Within these limitations, our experience suggests that direct innominate artery cannulation is a simple, fast, safe, and effective method of administrating SACP during hypothermic circulatory arrest for patients with acute type A dissection.

## Data Availability

The datasets used or analyzed during the current study are available from the corresponding author on reasonable request.

## Abbreviations

SACP: Selective antegrade cerebral perfusion
ACP: Antegrade cerebral perfusion
RCP: Retrograde cerebral perfusion
